# Pelvic Synovial Sarcoma Clinically Masquerading as an Ovarian Malignancy

**DOI:** 10.31486/toj.22.0046

**Published:** 2023

**Authors:** Deepika Gupta, Aasma Nalwa, Deepak Vedant, Abhishek Malik, Amit K. Gupta

**Affiliations:** Department of Pathology and Laboratory Medicine, All India Institute of Medical Sciences, Jodhpur, Rajasthan, India

**Keywords:** *Adenocarcinoma*, *ovarian neoplasms*, *pelvis*, *sarcoma–synovial*

## Abstract

**Background:** Synovial sarcoma, a malignant mesenchymal tumor, commonly involves the extremities and is rarely found in the pelvis. Cytology with a biphasic pattern can suggest the diagnosis of synovial sarcoma.

**Case Report:** A 32-year-old female presented with complaints of abdominal distension. She was initially evaluated in another hospital where she underwent ultrasound-guided fine needle aspiration cytology (FNAC) of the abdominal mass. The mass was diagnosed as ovarian adenocarcinoma, and the patient underwent 3 cycles of chemotherapy. After 3 months, she was referred to our institute for surgical excision of the mass. Contrast-enhanced computed tomography of the abdomen suggested a well-circumscribed, heterogeneous, solid-cystic mass in the left adnexal region measuring 13.9 × 10 × 9.1 cm and compressing the adjacent structures. No previous radiologic investigations were available. We reviewed the FNAC slide from the outside hospital and made a preliminary diagnosis of biphasic synovial sarcoma. The patient underwent debulking surgery consisting of panhysterectomy with excision of the pelvic mass. Microscopic examination of the pelvic mass showed a biphasic tumor composed of epithelial and mesenchymal components, suggestive of synovial sarcoma. The immunohistochemistry profile supported the morphologic diagnosis. Bilateral ovaries were unremarkable. The patient received 4 cycles of adjuvant chemotherapy and is presently asymptomatic.

**Conclusion:** Although primary pelvic synovial sarcoma is a rare occurrence, this case illustrates that synovial sarcoma can be diagnosed or at least suspected on FNAC. Because of the importance of adjuvant chemotherapy, synovial sarcoma must be high on the list of differential diagnoses of high-grade intra-abdominal masses.

## INTRODUCTION

Synovial sarcoma is a soft tissue sarcoma with specific clinicopathologic and genetic features that can occur anywhere in the body. The most common site is the extremities near the large joints (70%).^1^ Primary pelvic synovial sarcoma is exceptionally rare; only a few cases have been reported in the literature,^2-12^ and we could not determine the exact incidence. Histologically, synovial sarcoma has 2 main subtypes: (1) biphasic with both epithelial and spindle cell components, and (2) monophasic with predominantly spindle cells.^1^ Fine needle aspiration cytology (FNAC) is a well-accepted, minimally invasive procedure and an effective method of diagnosing benign and malignant soft tissue tumors of the extremities and deep body cavities. Precise morphologic diagnosis and grading of sarcomas by FNAC is problematic; however, biphasic synovial sarcomas have been successfully diagnosed on aspiration.^13^ Synovial sarcomas often display morphologic features that overlap with a variety of other spindle cell sarcomas and carcinomas such as malignant peripheral nerve sheath tumor, leiomyosarcoma, solitary fibrous tumor, Ewing sarcoma/primitive neuroectodermal tumor, fibrosarcoma, sarcomatoid carcinoma, Müllerian adenosarcoma, and sarcomatoid mesothelioma.^2,3^

We present a case of pelvic synovial sarcoma that was originally diagnosed as an ovarian malignancy.

## CASE REPORT

A 32-year-old female presented at an outside hospital with complaints of abdominal distension for 1 month. She underwent ultrasound-guided FNAC of the abdominal mass, and it was diagnosed as adenocarcinoma of the ovary. She received 3 cycles of chemotherapy (paclitaxel 280 mg + carboplatin 550 mg).

After 3 months, the patient was referred to our institute for surgical excision. At the time of admission, liver and kidney function tests were within normal range. A complete blood count revealed hemoglobin 10.7 g/dL (reference range, 11.5-15 g/dL), total leukocyte count 8,870/μL (reference range, 4,000-11,000/μL), and platelet count 567 × 10^9^/L (reference range, 150-400 × 10^9^/L). The patient was nonreactive for hepatitis B, hepatitis C, HIV-1, and HIV-2.

Contrast-enhanced computed tomography (CT) of the abdomen showed a well-circumscribed, heterogeneous, solid-cystic mass in the left adnexal region measuring 13.9 × 10 × 9.1 cm and compressing the adjacent structures.

**Figure 1.**
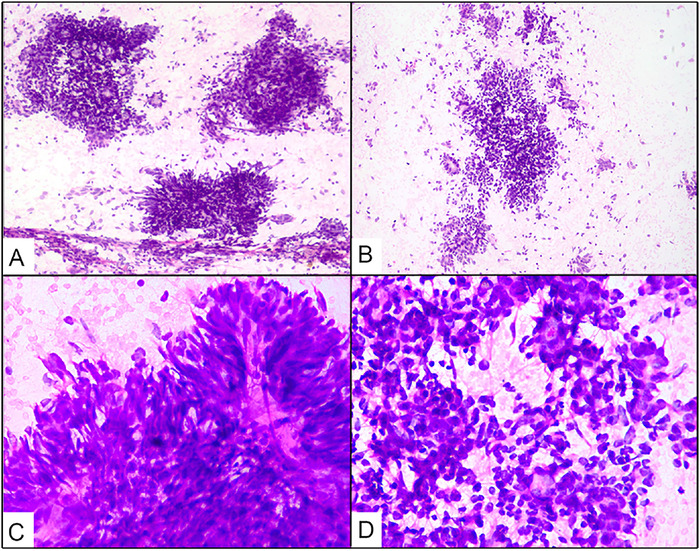
(A, B) Loosely cohesive clusters and numerous scattered bland-looking spindle cells (hematoxylin and eosin stain [H&E], magnification ×100). (C) Spindle cells have fine chromatin, inconspicuous nucleoli, and elongated delicate cytoplasm (H&E, magnification ×400). (D) Epithelioid-like cells form acinar-like structures (H&E, magnification ×400).

One hematoxylin and eosin stained slide of the FNAC of the abdominal mass from the outside hospital was received for review. The smear was cellular, displaying loosely cohesive clusters, as well as numerous scattered bland-looking spindle cells with fine chromatin, inconspicuous nucleoli, and elongated delicate cytoplasm (Figures 1A, 1B, and 1C). On high-power evaluation, epithelioid-like cells forming acinar-like structures were also identified (Figure 1D). Mitoses were infrequent. The cytomorphology suggested the diagnosis of biphasic spindle cell neoplasm resembling a synovial sarcoma. No immunostaining was performed on the smear or cell block, as only one stained smear was received for review. No FNAC was performed at our institute.

**Figure 2.**
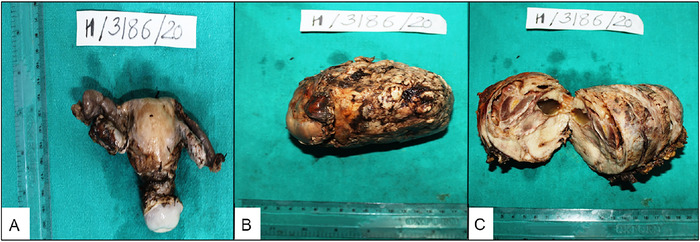
(A) Total hysterectomy specimen with bilateral adnexa. The ovaries are normal size. (B) The pelvic mass measured approximately 11 × 8.5 × 8 cm. (C) On the cut section, the pelvic mass was solid-cystic with variable-sized cysts filled with gelatinous fluid.

The patient underwent debulking surgery consisting of panhysterectomy (Figure 2A), excision of the pelvic mass with omentectomy, and mesenteric lymph nodal dissection. The pelvic mass measured 11 × 8.5 × 8 cm with part of the capsule at one end (Figure 2B). On serial sectioning, the mass was solid-cystic; the cysts were filled with gelatinous fluid (Figure 2C), and the largest cyst measured 3 × 2 × 1 cm. Uterus and cervix were unremarkable grossly; however, the uterus showed an intramural leiomyoma. Bilateral ovaries and fallopian tubes were unremarkable grossly and separate from the tumor.

**Figure 3.**
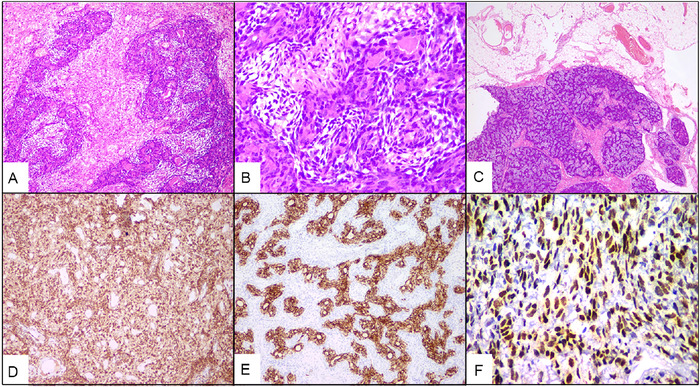
(A) Biphasic tumor composed of epithelial and mesenchymal components (hematoxylin and eosin stain [H&E], magnification ×100). (B) Epithelial component forming glands with interspersed spindle-shaped mesenchymal cells (H&E, magnification ×400). (C) Omentum showing tumor deposits (H&E, magnification ×20). Tumor cells showing strong immunoreactivity for (D) BCL2 (immunohistochemistry [IHC], magnification ×100), (E) cytokeratin (IHC, magnification ×100), and (F) TLE1 (IHC, magnification ×400).

Light microscopic examination of the pelvic mass showed a biphasic tumor composed of epithelial and mesenchymal components (Figure 3A). The epithelial component formed glands with interspersed spindle-shaped mesenchymal cells (Figure 3B), and the epithelial cells had coarse chromatin and moderate cytoplasm. The mesenchymal component was composed of spindle cells arranged in fascicles with uniform bland nuclei and pale, ill-defined cytoplasm in a background of loose collagenous stroma with large areas of necrosis and rare mitosis. Tumor deposits were seen in the omentum (Figure 3C). On immunohistochemistry, tumor cells were immunopositive for BCL2 (Figure 3D), cytokeratin (Figure 3E), and TLE1 (Figure 3F) and were negative for PAX8. Bilateral ovaries were uninvolved and free of tumor. Based on the morphologic and immunohistochemical findings, biphasic synovial sarcoma was diagnosed. Molecular diagnosis was not performed because of financial constraints.

The patient received 4 cycles of adjuvant chemotherapy (cisplatin 120 mg/m^2^ for 1 day, followed 1 week later by ifosfamide 2 g/m^2^ for 5 days and doxorubicin (Adriamycin) 30 mg/m^2^ for 3 days) and was disease-free 21 months posttreatment on follow-up. The patient is being followed every 5 to 6 months for the next 3 to 5 years, and the surveillance regimen includes clinical history and CT scan.

## DISCUSSION

Soft tissue sarcomas account for <1.5% of all malignant neoplasms in adults.^14^ An array of soft tissue sarcomas with musculoskeletal histology can occur in the abdomen and pelvis, posing challenging problems because of their complex anatomy and initial presentation as large masses with extensive invasion. Synovial sarcoma represents 5% to 10% of all soft tissue sarcomas.^15,16^ Of these synovial sarcomas, 85% to 95% occur in the extremities, with a predilection for the lower extremities, followed by 5% to 10% in the head and neck region and 5% in the trunk, including the chest wall and abdomen.^14^

The specific cell origin is unknown; however, an unknown multipotent stem cell capable of mesenchymal and/or epithelial differentiation but lacking synovial differentiation may be the culprit.^17^ Synovial sarcoma affects young adults without any sex predilection. Deshmukh et al reported a mild male predominance of 1.2:1^18^; in contrast, a slight female predominance has been reported by others,^19^ whereas no significant difference in sex distribution was seen in a series of 672 patients conducted over a period of 10 years.^15^ Symptoms vary with the site and size of the tumor.

Descriptions of cytomorphologic features of synovial sarcoma are based on case reports and case series, with only an occasional study investigating the cytologic criteria for forming a reliable diagnosis on FNAC. One such investigation was carried out by the Scandinavian Sarcoma Group that studied 104 synovial sarcomas.^20^ Typically, aspirates were cellular and composed of a mixed population of cohesive tissue fragments and scattered cells, along with numerous bare nuclei.^20^ Mesenchymal cells were small to medium-sized and round to spindle-shaped, with round or fusiform nuclei with bland nuclear features and scant tapering cytoplasm.^20,21^ Mitosis and mast cells could be seen in tissue fragments.^20^ In some cases, the mesenchymal component was admixed with epithelial-like structures resembling pseudoacinar formations (acini-like).^3^

FNAC is an effective diagnostic tool for preoperative evaluation and assessment of recurrence and/or metastasis in soft tissue sarcomas. Sensitivity and specificity >90% are seen with experienced hands, similar to frozen section interpretation rates.^22^

Kilpatrick et al evaluated the effectiveness of FNAC in subtyping soft tissue sarcomas by analyzing the cytomorphologic features in 73 consecutive aspirates from 67 patients and subgrouping them into pleomorphic cell (19 cases), small round cell (18 cases), spindle cell (18 cases), myxoid tumor (10 cases), epithelioid/polygonal cell (7 cases), and well-differentiated liposarcoma (1 case).^21^ In the spindle cell group, 7 cases were synovial sarcomas that were diagnosed on histology. Of these 7 cases, 6 were correctly diagnosed on cytology. One case was reported as spindle cell neoplasm that on histology was diagnosed as monophasic synovial sarcoma, suggesting that the presence of an epithelial cell component is necessary for the diagnosis of synovial sarcoma.

Nakamura et al studied 90 consecutive patients with pelvic soft tissue sarcomas over 23 years and found 6 patients with synovial sarcoma (6.7%), concluding that high-grade tumors and positive surgical margins represent a high-risk group for local recurrence.^23^ Fisher et al reviewed 300 cases of synovial sarcoma, of which 3 were pelvic and 8 were retroperitoneal, and concluded that pelvic tumors metastasize distantly.^2^ In contrast, retroperitoneal tumors remain confined to the abdomen and do not metastasize remotely.^2^

In the pelvis, synovial sarcomas need to be distinguished from gynecologic malignancies such as carcinosarcoma and soft tissue tumors such as malignant peripheral nerve sheath tumor, leiomyosarcoma, solitary fibrous tumor, fibrosarcoma, sarcomatoid mesothelioma, sarcomatoid carcinoma, Müllerian adenosarcoma, and Ewing sarcoma.^24^ Malignant peripheral nerve sheath tumor is an important differential of monophasic synovial sarcoma on cytology. The Table shows the differential diagnosis of synovial sarcoma in the pelvis.^2,18,20,21,24-32^

**Table.** Differential Diagnosis of Synovial Sarcoma in the Pelvis

Tumor	Cytomorphologic Features	Immunohistochemistry Profile and Cytogenetics
Malignant peripheral nerve sheath tumor	Hypocellular and hypercellular areas with cells showing wavy nuclei, nuclear buckling/kinking/twisting, and nuclear pseudoinclusions^26^	S100 protein^27^, SOX10
Leiomyosarcoma	Spindle-shaped cells arranged in intersecting fascicles having blunt cigar-shaped nuclei with paranuclear vacuolations and abundant eosinophilic cytoplasm^26^	Smooth muscle actin, desmin, and h-Caldesmon; can show focal expression of epithelial membrane antigen and keratin in a dot-like pattern^27^
Solitary fibrous tumor	Spindle-shaped cells with ovoid vesicular nuclei arranged in a patternless way, rare mitotic figures, varying amounts of collagenous stroma, prominent thin-walled hemangiopericytic vessels^26^	Diffuse expression of CD34,^26^ diffuse nuclear expression of STAT6^26^; one-third express AE1/AE3; nuclear expression of FLI1
Ewing sarcoma/primitive neuroectodermal tumor	Isolated or sparsely clustered round to oval uniform small cells with central nuclei, rosette formation^28^	Positive periodic acid-Schiff stain, strong membranous immunoreactivity for CD99,^18^ caveolin-1^26^ Cytogenetic analysis for t(11;22) for confirmation of Ewing sarcoma^14^
Fibrosarcoma	Diagnosis of exclusion, spindle cells arranged in a herringbone pattern, can be pleomorphic, produces intercellular collagen^2^	Negative for cytokeratin and epithelial membrane antigen^2^
Sarcomatoid carcinoma and Müllerian adenosarcoma	More pleomorphic than synovial sarcoma^2^	Diffuse cytokeratin positivity^2^
Sarcomatoid mesothelioma	Larger and less uniform nuclei,^2^ single cells and loose aggregates, short spindle-shaped cells without microvilli^30^	Calretinin, podoplanin (D2-40), WT1, CK5/6, mesothelin^31^
Synovial sarcoma	Mixed population of cohesive tissue fragments and scattered cells, tissue fragments contain thin branching capillaries, mitosis and mast cells present, sometimes admixed with epithelial-like structures^20,21^	Epithelial membrane antigen, CK7,^24^ CK19,^24^ BCL2 (nearly 100% of synovial sarcoma cases), CD56 (nearly 100% of synovial sarcoma cases),^25^ CD99 (60% of synovial sarcoma cases),^20^ S100P (30% of synovial sarcoma cases),^20^ TLE1(most sensitive and specific)^29^ Cytogenetic analysis for detection of t(X;18)(p11.2;q11.2) translocation for confirmation of synovial sarcoma^24,28,32^

Immunohistochemically, the spindle cell population of synovial sarcoma can show focal or widespread positivity for epithelial membrane antigen (most sensitive) and for CK7 and CK19, which are nearly 100% specific.^24^ Hartel et al found that BCL2 and CD56 reach nearly 100% positivity in cases of primary pulmonary and mediastinal synovial sarcoma,^25^ and approximately 60% of synovial sarcoma are positive for CD99.^20^ TLE1 is the most sensitive and specific marker for synovial sarcoma.^29^

Surgery is the treatment mainstay; however, marginal clearance is difficult to achieve for tumors in the pelvic region. Synovial sarcoma is relatively chemosensitive to standard first-line treatment (combination treatment with doxorubicin and ifosfamide is a preferred option) and second-line treatment (pazopanib and trabectedin).^24^ The use of newer drugs such as receptor tyrosine kinase inhibitors, epigenetic modifiers, and immunotherapy is under evaluation, and according to Desar et al, DNA damage response inhibitors should also be tested in clinical studies.^24^ Local recurrence and propensity for distant metastasis are seen in approximately 50% of cases.^24^ The t(X;18) (p11.2;q11.2) translocation, resulting in SS18-SSX (SSX1, SSX2, and SSX4) fusion of oncogenes, is seen in 90% of cases.^24-29^

A multidisciplinary approach combining clinical consideration with careful scrutiny of morphologic features can help make the diagnosis. The present case illustrates that synovial sarcoma can be diagnosed or at least suspected on FNAC.

## CONCLUSION

Primary pelvic synovial sarcoma is a rare entity. The importance of adjuvant chemotherapy has been identified, so synovial sarcoma must be high on the list of differential diagnoses of high-grade intra-abdominal masses, and FNAC can be helpful in preoperative diagnosis.

## Notes

*This article meets the Accreditation Council for Graduate Medical Education and the American Board of Medical Specialties Maintenance of Certification competencies for Patient Care and Medical Knowledge.*
